# Social media addiction: associations with attachment style, mental distress, and personality

**DOI:** 10.1186/s12888-024-05709-z

**Published:** 2024-04-15

**Authors:** Christiane Eichenberg, Raphaela Schneider, Helena Rumpl

**Affiliations:** Faculty of Medicine, Institute of Psychosomatics, Sigmund Freud Private University, Freudplatz 3, Vienna, 1020 Austria

**Keywords:** Social media addiction, Attachment, Personality, Internet related addiction, Mental distress, Insecure attachment style

## Abstract

**Background:**

Social media bring not only benefits but also downsides, such as addictive behavior. While an ambivalent closed insecure attachment style has been prominently linked with internet and smartphone addiction, a similar analysis for social media addiction is still pending. This study aims to explore social media addiction, focusing on variations in attachment style, mental distress, and personality between students with and without problematic social media use. Additionally, it investigates whether a specific attachment style is connected to social media addiction.

**Methods:**

Data were collected from 571 college students (mean age = 23.61, *SD* = 5.00, 65.5% female; response rate = 20.06%) via an online survey administered to all enrolled students of Sigmund Freud PrivatUniversity Vienna. The Bergen Social Media Addiction Scale (BSMAS) differentiated between students addicted and not addicted to social media. Attachment style was gauged using the Bielefeld Partnership Expectations Questionnaire (BFPE), mental distress by the Brief Symptom Inventory (BSI-18), and personality by the Big Five Inventory (BFI-10).

**Results:**

Of the total sample, 22.7% of students were identified as addicted to social media. For personality, it was demonstrated that socially media addicted (SMA) students reported significantly higher values on the neuroticism dimension compared to not socially media addicted (NSMA) students. SMA also scored higher across all mental health dimensions—depressiveness, anxiety, and somatization. SMA more frequently exhibited an insecure attachment style than NSMA, specifically, an ambivalent closed attachment style. A two-step cluster analysis validated the initial findings, uncovering three clusters: (1) secure attachment, primarily linked with fewer occurrences of social media addiction and a lower incidence of mental health problems; (2) ambivalent closed attachment, generally associated with a higher rate of social media addiction and increased levels of mental health problems; and (3) ambivalent clingy attachment, manifesting a medium prevalence of social media addiction and a relatively equitable mental health profile.

**Conclusions:**

The outcomes are aligned with previous research on internet and smartphone addiction, pointing out the relevance of an ambivalent closed attachment style in all three contexts. Therapeutic interventions for social media addiction should be developed and implemented considering these findings.

## Introduction

Digital media have become ubiquitous. As of April 2023, 5.18 billion people worldwide use the Internet [1]. On average, global Internet users spend 6 h and 43 min online daily [2]. In 2023, social media platforms engage 4.8 billion users worldwide, a significant rise from 2.46 billion in 2017 [1, 2]. These users spend an average of 2 h and 25 min on social networks each day and have, on average, 8.9 social media accounts [2]. Smartphones, now an essential device for many, are especially popular among the youth. Specifically, teenagers aged 14 to 24 access their phones approximately 214 times daily [3]. While social media networks have grown in importance, they also introduce challenges. Issues such as social media fatigue manifest in negative emotional responses like burnout, exhaustion, and frustration during social network activities [4]. Another possible negative consequence of social media activity is addictive behavior that is reported prior in the context of internet addiction.

## Classification and definition of social media addiction

Digital media addictions, with a particular emphasis on social media addictions, are increasingly prevalent in psychotherapy, especially among younger demographics [5, 6]. The concern for social media addiction is heightened among females, who show a higher propensity towards this addiction [7, 8]. Despite its growing prevalence, social media addiction is yet to be fully acknowledged in diagnostic classification systems. The term “addiction” is therefore only used in this context for the sake of simplicity, as it is not yet officially recognized. The concept of ‘behavioral addiction,’ which characterizes excessive, rewarding behaviors leading to psychological addiction symptoms [9], is applicable here, though social media addiction still lacks distinct recognition in diagnostic manuals like the ICD and DSM. This gap highlights the need for more comprehensive research and understanding.

Prior research conforms mainly to differentiate between generalized and specific internet addictions [10–13]. The first means a multidimensional misuse of the internet using multiple internet functions, whereas the ladder aims a sole specific internet function (e.g., gaming, gambling, social media etc.) [13, 14]. Social Media Addiction, encompassing variants like Facebook addiction and general addictive use of social networking sites (SNSs), is characterized as a maladaptive psychological dependency on SNSs, leading to behavioral addiction symptoms [15–17]. Currently, Social Media Addiction assessment relies on questionnaires like the Bergen Social Media Addiction Scale (BSMAS [18]),, which is momentarily the most widely used tool and applies criteria such as salience, mood modification, tolerance, withdrawal, conflict, and relapse [19] to evaluate addictive behaviors [10].

## Prevalence rates and mental stress correlations of social media addiction

Data regarding the prevalence of social media addiction indicate a range between 1% and 18.7% [20]. However, the accuracy of these rates is debated. Cheng et al. [21] suggest that estimates of social media addiction are often either under- or overestimated. Their recent meta-analysis revealed prevalence rates ranging from 0 to 82%, a wide disparity stemming from differing theoretical frameworks and measurement instruments. Depending on the strictness of the classification system used, the researchers identified three mean prevalence benchmarks: 5%, 13%, and 25%. Frequently, individuals with problematic social media use also grapple with other mental health issues. Depression [20, 22] and social anxiety [23] are commonly co-occurring disorders, as are challenges related to self-esteem (ibid.). Particularly, young women often feel dissatisfied with their bodies due to social media engagement. The frequent exposure to manipulated and idealized images of models or influencers fuels a comparison culture. As a result, many young women develop a desire to alter their appearance [24]. The number of “likes” they receive on platforms becomes a proxy for their self-worth, heavily influencing their self-esteem [25]. Several studies highlight that young adults spending over two hours daily on social media tend to exhibit higher rates of anxiety, depression, and sleep disturbances.

## Personality traits and social media addiction

The personality trait neuroticism, and the “fear of missing out” or FOMO [26], have been identified as predictors of Social Media Addiction [27]. Conversely, extraversion’s link to social media use is debated. While some evidence suggests extraversion is not a significant factor [28], other research indicates extraverted individuals are more prone to social media use and potential addiction. Kuss & Griffiths [29] offer a more nuanced view in their literature review. According to them, extraverted individuals might use social media to augment their social interactions, i.e. they use social media in a positive manner to expand opportunities to interact with others in more ways. Introverted users, on the other hand, use social media to compensate for a perceived social deficit. For them, using social media is a way to connect with others in a way that they feel is not sufficiently possible in real life.

## Attachment styles and social media addiction

Extensive research has been conducted on the association between insecure attachment and substance addictions [30, 31]. The attachment system, which comprises secure, insecure, and disorganized categories, is a biologically and evolutionarily rooted motivational and behavioral system that operates through attachment figures [32]. Schuhler et al. [33] proposed a model elucidating the link between internet addiction and attachment, suggesting that addictive behaviors may arise as a means to compensate for attachment issues. From this perspective, digital addiction represents a flawed attempt to address early attachment deficiencies [33, 34]. In a related vein, Brisch [35] introduced a model that positions the ‘reference object’ as central to the understanding of addictions. According to this model, the primary function of social media addiction isn’t to escape negative emotions, as is often the case with substance addictions. Instead, it’s seen as an excessive digitally-mediated social behavior aiming to substitute for insecure attachments. Supporting this, Eichenberg et al. [34] showed that insecure attachment style is correlated with problematic smartphone usage and problematic internet usage [36]. Notably, an ambivalently attached style was identified as particularly relevant in both contexts. A plethora of studies showed a link between social media addiction and attachment in general [37–43]. But the question arises whether the specific attachment style as has shown relevant for internet and smartphone addiction will also be prominent for social media addiction.

## Research objectives and questions

### Attachment

The primary objective of this study is to explore whether an insecure attachment style correlates with addictive social media use, and to pinpoint which specific style is most relevant. While research has identified an ambivalent closed insecure attachment style as being significant in the context of internet and smartphone addiction, a detailed examination specific to social media addiction remains lacking.

Moreover, this study seeks to gather further information regarding the still emerging psychopathology, specifically focusing on the personality traits neuroticism and extraversion, as well as mental stress.

### Mental health

The research questions will be, whether social media addicted students report higher levels of depression, anxiety, and somatization.

### Personality

Further, it will be explored whether neuroticism and extraversion influence an individual’s susceptibility to social media addiction.

## Methods

### Recruitment

A comprehensive survey (*N* = 2846, response rate = 20.06%) was created with the SoSci Survey online survey tool [44] and was conducted among students at the Sigmund Freud PrivatUniversität in Vienna, Austria. The data collection took place from January to March 2021, resulting in a final sample of 571 respondents. To distribute the online questionnaire, the Study Service Centers from the faculties of psychology, psychotherapy, law, and medicine were approached. They were requested to email the link to the questionnaire, accompanied by a pre-written invitation text, to all actively enrolled students at the Sigmund Freud PrivatUniversität Vienna. Once the participants provided informed consent and completed the survey, they were redirected to a debriefing page. This page detailed the study’s objectives and offered the contact information of the researchers, in case the participants sought support related to the survey topics or had additional inquiries. The survey received approval from the Ethics Commission of the Faculty of Psychotherapy Science and the Faculty of Psychology of the Sigμund Freud University PrivatVienna. Recognizing the sensitive nature of the topic, paramount emphasis was placed on safeguarding the confidentiality of participants’ responses. Furthermore, participants had the liberty to opt out of the study at any juncture. Should they wish to have their data expunged, they could simply reach out to a researcher via email, referencing an unique anonymized code. This would enable the researcher to identify and delete the participant’s anonymized data.

### Survey structure

The survey, created using Sosci-Survey, began with a brief that outlined the research rationale and the survey’s objectives. Participants affirmed their agreement with the study’s privacy policy through a checkbox.

**Section 1** asked about socio-demographic factors, including age, gender, and study subject. Subsequently, it touched upon matters related to social media:

*Services most used*: Participants identified which social media services they frequently use, answered dichotomously (yes/no).

*Usage frequency*: Choices ranged from “less than 30 minutes” to “more than four hours per day” on a seven-point scale.

*Social Media Importance*: Participants rated from “very significant” to “not significant” on a four-point scale.

*Purposes of Use*: Employing a five-point scale, respondents indicated frequency, ranging from 1 (“never”) to 5 (“several times a day”).

*Perceived downsides*: Participants assessed their sentiments on a five-point scale from 1 (“not true at all”) to 5 (“completely true”).

In light of evidence suggesting a discrepancy between objective and self-reported usage time—where users often overestimate their screen time [45]—the survey did not deploy open-ended questions concerning usage duration. Instead, participants were presented with predefined categories to streamline their responses.

**Section 2** incorporated standardized questionnaires to examine further social media addiction, mental distress, personality traits, and attachment styles.

### Bergen social media addiction scale BSMAS [18]

The Bergen Social Media Addiction Scale (BSMAS) [18] categorizes users into two groups: those addicted to social media and those not addicted. All six items pertain to one’s experience with social media over the past 12 months. It employs a five-point scale, ranging from 1 (“very rarely”) to 5 (“very often”). The scale asks at the beginning of each item “How often during the last year have you…” and continues with “…spent a lot of time thinking about social media or planned use of social media?” (i.e., salience) or “…become restless or troubled if you have been prohibited from using social media?” (i.e., withdrawal). A higher BSMAS score indicates a heightened risk of social media addiction. As suggested by a substantial Hungarian study involving 6000 adolescents [20], a cutoff score of 19 out of 30 was adopted. The scale was repeatedly reported with high internal consistency, e.g., α = 0.97 [46] and α = 0.82 (at baseline) plus α = 0.86 (at follow-up) [10]. Chen et al. [10] confirm the single-factor structure of the scale, report only medium correlations with scales close to the construct (SABAS/smartphone addiction, IGDS-SF9/internet gaming disorder, *r* =.06 and 0.42), and showed invariance across three months among young adults. They presented a good test–retest reliability after three months (*ICC* = 0.86, *p* <.001).

### Brief symptom inventory BSI-18 [47]

The BSI-18 is a brief, reliable instrument for assessing mental stress. It contains the three subscales somatization, depression, and anxiety, comprising 6 items, as well as the Global Severity Index (GSI) including all 18 items. Response format of the 18 items is a five-point scale (0=”not at all” to 4=”very strong”). The scale asks at the beginning of a symptoms list: “How much have you had within the past 7 days…”. Examples for the symptoms on this list are “Nausea or upset stomach” for somatization, “Feelings of worthlessness” for depression”, and “Spells of terror or panic” for anxiety. The BSI-18 is the newest and shortest of the multidimensional versions of the Symptom Checklist 90-R. The BSI-18 assesses validly mental stress in both normal population [48] and clinical populations [49]. Confirmatory analyses confirm the three-factor structure [48]. Franke et al. [49] report good internal consistencies of the scales fear of rejection (BSI-18 (α (somatization) = 0,79, α (depression) = 0,84, α (anxiety) = 0,84, α (GSI) = 0,91).

### Big five inventory BFI-10 [50]

The questionnaire is based on the Big Five personality traits model, also called OCEAN model that is the most widely used model for describing overall personality [51]. Theoretical background is the sedimentation hypothesis that assumes that every personality trait must be represented in language and, therefore, factor analyses were used to find universal personality dimensions [52]. Multiple analyses by various researchers resulted repeatedly in the OCEAN model, which consists of the five dimensions agreeableness, neuroticism, conscientiousness, openness to experience, and extraversion. The BFI-10 [50] contains 10 items, two for each of the five dimensions. The scale asks, “How well do the following statements describe your personality?” and starts a list of attitudes with “I see myself as someone who…“. Example answers are: “…does a thorough job” (i.e., conscientiousness) or “…is outgoing, sociable” (i.e., extraversion). Respondents answered a five-point rating scale from “does not apply at all” (1) to “applies completely” (5) for each item. Rammstedt und John [50] report moderate test–retest reliability after 6 weeks in a student sample (agreeableness: *rtt* = 0.58, neuroticism: *rtt* = 0.74, conscientiousness: *rtt* = 0.77, openness to experience: *rtt* = 0.72, extraversion: *rtt* = 0.84). In a representative sample, however, the retest coefficients are lower overall ranging from (*rtt* =.62) for openness to experience to (*rtt* =.49) for neuroticism [51]. Rammstedt et al. [51] report sufficient construct validity correlating the BFI-10 with the NEO-PI-R and factorial validity by conducting principal component analyses on a representative sample.

### Bielefeld questionnaire on partnership expectations BFPE [53]

The BFPE operationalizes attachment styles of adults by recording self-reports on three scales: conscious need for care (8 items), fear of rejection (11 items), and readiness for self-disclosure (11 items) [53]. Example items are: “Knowing myself as I do, I can hardly imagine that my partner will appreciate me” (i.e., fear of rejection), “I prefer to talk with my partner about facts rather than about feelings” (i.e., readiness for self- disclosure), and “It’s important for me that my partner thinks of me often, even when we are not together” (i.e., conscious need for care). The first of the 31 items serves as an icebreaker item and is not evaluated. The degree of expression of each item is indicated on a 5-point scale (1= “does not apply at all” to 5 = “applies exactly”). From the aggregate scores of these scales, one of five attachment styles can be determined: secure, two variations of ambivalent/anxious (closed and clinging), and two variations of the avoidant style (closed and conditionally secure). For simplification purposes, these styles can be dichotomized into two primary categories: secure (which includes both secure and conditionally secure types) and insecure (encompassing avoidant-closed, ambivalent-clingy, and ambivalent-closed types). These distinct attachment styles emerged originally from cluster analysis research [53]. Höger and Buschkämper [53] report good internal consistencies of the scales fear of rejection (Cronbach’s α = 0.88), readiness for self-disclosure (Cronbach’s α = 0.89), and conscious need for care (Cronbach’s α = 0.77). The split-half reliabilities calculated according to Guttman and Spearman-Brown are also similarly good for the three scales (fear of rejection = 0.91, readiness for self-disclosure = 0.89, and conscious need for care = 0.77). A validation is based on a German translation of the “Adult Attachment Scale” (AAS [54]),.

### Statistical analysis

The Statistical Package for the Social Sciences Program (SPSS version 27) was used for data input, processing, and statistical analyses. The participants were divided into social media addicted (SMA) and not addicted (NSMA) using the cut-off score according to Bányai et al. [20]. Additionally, the percentage of social media dependent students has been calculated. To evaluate differences between SMA and NSMA in social media usage, Mann-Whitney U tests for two independent samples were analyzed for differences in downsides of social media and usage purposes, and chi-square tests for differences in social media services, usage frequency, and social media importance, as the corresponding data were not normally distributed. Based on the data obtained with the BFPE, participants were allocated (see above) to the five attachment styles “secure,” “conditionally secure,” “ambivalent clingy,” “ambivalent closed,” and “avoidant closed.” Subsequently, the five attachment styles were dichotomized into the variables “secure” and “insecure” attachment styles. Subsequently, the five attachment styles were dichotomized into the variables “secure” and “insecure” attachment styles. Finally, using the chi-square tests, attachment styles and social media addiction were tested for significance differences. While chi-square tests provide valuable insights into individual associations, a two-step cluster analysis was conducted to gain a comprehensive understanding of how these variables collectively group participants. Two-step cluster analysis was chosen due to its capacity to handle both continuous and categorical variables. The number of clusters was determined based on the Schwarz Bayesian Criterion (BIC), and the selected model was further validated by examining the silhouette measure of cohesion and separation. Since gender and age are variables that could influence social media addiction, they were included in the cluster analysis to investigate their distribution over the resulting clusters. To maintain robustness of analyses, the non-binary gender category was omitted due to very small case number.

## Results

### Sample

The total sample (*N* = 571) consisted of 65.5% female students (*n* = 374) 33.3% male students (*n* = 190), and 1.2% those who did not wish to be defined by these two genders (*n* = 7). Participants were between 18 and 60 years old (*M* = 23.61 years, *SD* = 5.00, median = 23, modus = 22). The distribution of study subject was the following: medicine (*n* = 344, 59.7%), psychology (*n* = 121, 21.0%), psychotherapy (*n* = 79, 13.7%), and law (*n* = 32, 5.6%) (some students studied two subjects).

### Social media addiction

A total of 131 people (22.7% of the total sample) could be classified as addicted to social media. In addition, it was also relevant how genders were distributed between the two groups. Of the total number of participants classified as addicted participants (*N* = 131), 79.39% were female, 19.08% male, and 1.53% non-binary. These values are to be contrasted with the group of not addicted (*N* = 440), in which 61.36% were female, 37.5% male, and 1.14% non-binary.

### Social media usage

Among the various social media platforms, “WhatsApp” was the predominant choice with 99.1% usage. It was trailed by “YouTube” at 91.2%, “Instagram” at 82.1%, “Facebook” at 66.9%, “Snapchat” at 63.7%, “Facebook Messenger” at 35.6%, “Pinterest” at 32.9%, and “Twitter” at 10.5%. In addressing frequency of use, a significant 91% indicated they access social media multiple times per day. Delving into the duration of daily usage: 12.8% were on for less than an hour, 25.6% used it for around an hour, 32.7% for two hours, 16.8% for three hours, and 12.1% devoted more than three hours. When participants were asked about the significance of social media, 8.9% viewed it as very important, 55.1% as important, 31.3% as less important, and a mere 4.7% as not important. Participants predominantly engaged with social media for “entertainment” (*M* = 4.17, *SD* = 1.05), staying “up to date” (*M* = 4.12, *SD* = 1.03), combating “boredom” (*M* = 3.94, *SD* = 1.22), maintaining “contact with family” (*M* = 3.86, *SD* = 1.2), and for “music” (*M* = 3.55, *SD* = 1.4). They also sought “inspiration (e.g., fashion, interior)” with a mean score of (*M* = 3.35, *SD* = 1.29). However, not all experiences were positive. Downsides associated with social media usage were led by “comparison with others” (*M* = 3.19, *SD* = 1.3), followed by “dissatisfaction with own body” (*M* = 2.55, *SD* = 1.38), “negative self-esteem in contact with influencers” (*M* = 2.23, *SD* = 1.32), and encountering “insults, intrusive behavior” (*M* = 1.88, *SD* = 1.3). Distinguishing between SMA and NSMA users, differences emerged in their consumption patterns (see for details Table 1). SMA users predominantly gravitated towards image-centric platforms such as “Instagram” (93.1% SMA vs. 78.9% NSMA) and “Pinterest” (46.6% SMA vs. 28.9% NSMA). Remarkably, SMA users expressed heightened concerns regarding the downsides “comparison with others” (*M* = 4.06, *SD* = 1.03 for SMA vs. *M* = 2.94, *SD* = 1.26 for NSMA), “dissatisfaction with own body (when viewing idealized bodies online)” (M = 3.45, SD = 1.34 for SMA vs. *M* = 2.28, *SD* = 1.28 for NSMA), and “negative self-esteem in contact with influencers” (*M* = 3.16, *SD* = 1.34 for SMA vs. *M* = 1.95, *SD* = 1.18 for NSMA). It became evident that SMA users faced enhanced negative repercussions, especially in terms of body perception when comparing themselves with images of others. In addition, SMA use social media as tool for more purposes than NSMA. Not addicted report here, to use social media only for contact with family and music equally often.

**Table 1:** Significance tests for differences between social media addicted and non addicted in general social media usage

Social media usage variable	χ²	*P*	Significance
Instagram	14	<.001	significant
Facebook	1.81	0.179	n.s.
Snapchat	9	0.003	n.s.
Facebook Messenger	2.39	0.122	n.s.
Pinterest	14.3	<.001	significant
Twitter	0.526	0.468	n.s.
Usage frequency	16.7	0.002	significant
Usage time spent	64.8	<.001	significant
Usage importance	67.6	<.001	significant
**Social media usage variable**	***U***	***p***	**Significance**
Entertainment	37897	<.001	significant
To be up to date	45460	<.001	significant
Boredom	40151	<.001	significant
Contact with family	29967	0.381	n.s.
Music	33423	0.004	n.s.
Inspiration	37998	<.001	significant
Comparison with others	43263	<.001	significant
Dissatisfaction with own body	42103	<.001	significant
Negative self-esteem in contact with influencers	43057	<.001	significant
Insults, intrusive behavior	40724	0.026	n.s.

*Due to Bonferroni correction, significance level is 0.002

### Attachment style

**Fig. 1:**
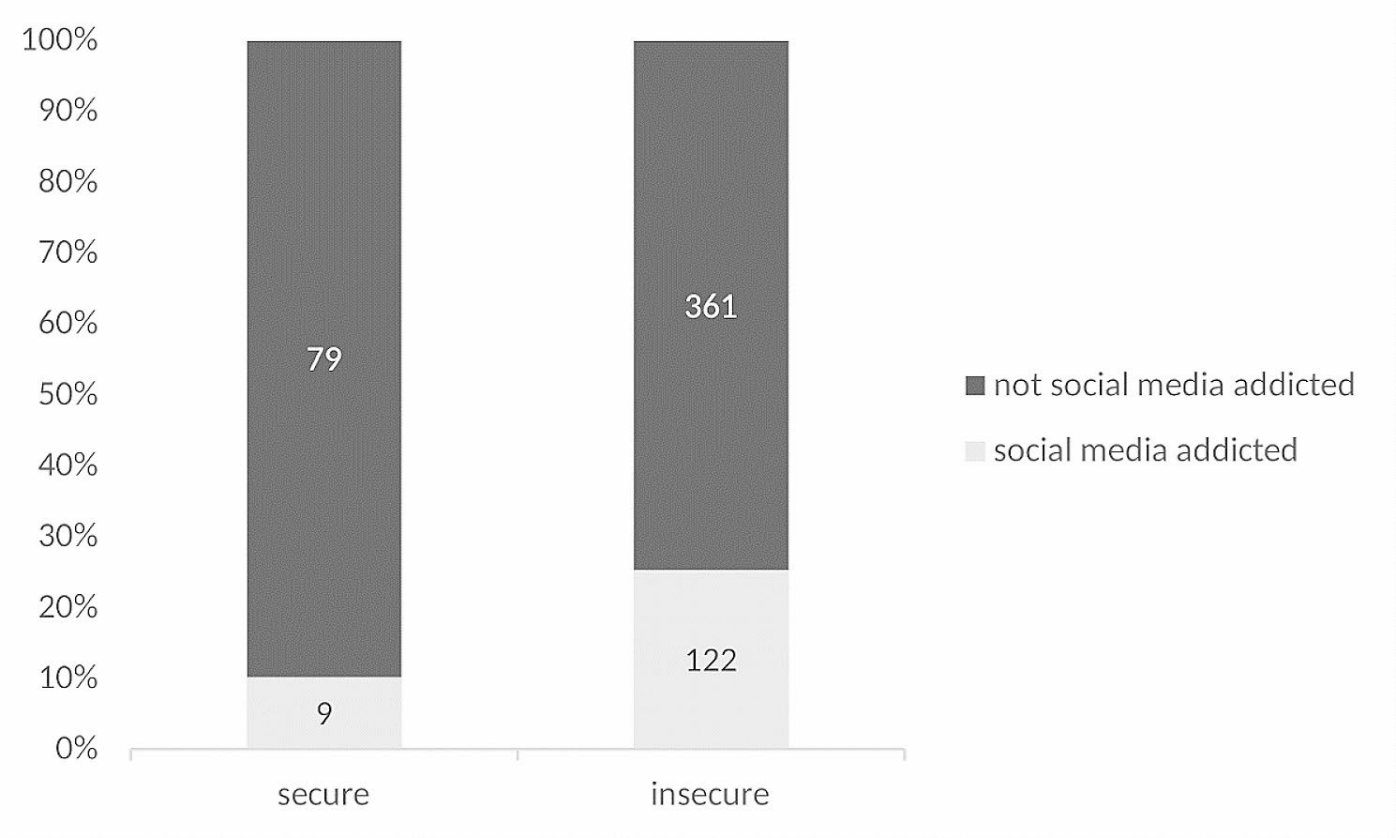
Relationship between attachment style and social media addiction. This stacked bar chart depicts the proportion of participants with ‘secure’ and ‘insecure’ attachment styles as determined by the Bielefeld Questionnaire on Partnership Expectations (BFPE). Attachment styles are defined by responses to three scales: conscious need for care, fear of rejection, and readiness for self-disclosure. These styles are subsequently dichotomized into ‘secure’ (including secure and conditionally secure styles) and ‘insecure’ (including avoidant-closed, ambivalent-clingy, and ambivalent-closed styles). Dark gray bars represent participants not addicted to social media, while light gray bars represent those with a self-reported addiction determined by the Bergen Social Media Addiction Scale (BSMAS). The numbers within the bars indicate the count of participants in each category

**Fig. 2:**
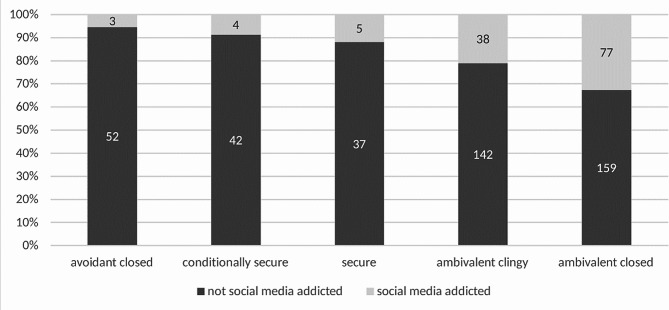
Distribution of five attachment styles and social media addiction. This bar chart visualizes the proportion of participants classified into five distinct attachment styles according to the Bielefeld Questionnaire on Partnership Expectations (BFPE) alongside their social media addiction status, as measured by the Bergen Social Media Addiction Scale (BSMAS). The attachment styles represented are ‘avoidant closed’, ‘conditionally secure’, ‘secure’, ‘ambivalent clingy’, and ‘ambivalent closed’. Dark gray bars indicate participants not identified as addicted to social media, while light gray bars represent those who meet the criteria for addiction according to the BSMAS. The numbers within the bars denote the count of participants corresponding to each category

Since 12 participants did not completely fill in the BFPE, the number of participants regarding attachment is 559. Frequencies and percentages of each attachment style can be seen in Table 2. A small part of the student population was securely bound (*n* = 88, 15.7%) with the biggest part being insecurely bound (*n* = 471, 84.3%). Secure attachment style (corrected residuals: 3.1) is related to a disproportionately higher number of NSMA and insecure attachment style (corrected residuals: 3.1) is related to a disproportionately higher number of SMA, *χ²*(1) = 9.28, *p =*.002, *C* = 0.13 (see Fig. 1, see Table 3). The five individual attachment styles differ in the frequency distribution of social media addiction, *χ²*(4) = 30.75, *p <*.001, *C* = 0.24, with avoidant closed (corrected residuals:3.2) having disproportionately more NSMA, ambivalent closed (corrected residuals: 4.8) having disproportionately more SMA, and conditionally secure (corrected residuals: 2.4) having disproportionately more NSMA (see Fig. 2). So, findings show that participants with social media addiction had a significant higher likelihood to have an ambivalent closed attachment style.

**Table 2:** Frequencies and percentages of each attachment style

Attachment style	*n*	%
Rather secure	46	8,2
Secure	42	7,5
Ambivalent clingy	180	32,2
Ambivalent closed	236	42,2
Avoidant closed	55	9,8
Total	559	100

**Table 3:** Social media addiction and dichotomized attachment style

	Insecure		Secure
Not social media addicted (NSMA)	*n* Expected	353 364	79 68
Social media addicted (SMA)	*n* Expected	118 107	9 20
Total	*n*	471	88

### Personality

Regarding extraversion, the total sample (*M* = 3.58, *SD* = 0.92, modus = 5, *Md* = 3.5) is slightly but significantly less open-minded than a norm sample having same age and education (*M* = 3.93, *SD* = 0.83, Rammstedt et al. 2012) (*t*(570)=-9.23, *p <*.001) and regarding neuroticism, the sample (*M* = 3.09, *SD* = 0.87, modus = 2.5, *Md* = 3) is significantly more neurotic than a comparable norm sample (*M* = 2.25, *SD* = 0.69, Rammstedt et al. 2012) (*t*(570) = 23.15, *p <*.001). Further, it was found that SMA (*M* = 3.40, *SD* = 0.85) scored significantly higher than NSMA (*M* = 3.00, *SD* = 0.85) on the dimension of neuroticism and thus could be classified as more emotionally unstable (*U* = 20636.50, *Z* = -5.02, *p <*.001). However, on the dimension of extraversion, SMA (*M* = 3.56, *SD* = 0.85) did not differ from NSMA (*M* = 3.58, *SD* = 0.94) (*U* = 28408.5, *Z* = − 0.25, *p =*.801).

### Mental distress

The total sample showed in comparison with a norm sample high levels of each of the three dimensions of depression (*M* = 4.18; *SD* = 4.52 vs. *M*^*norm*^=1.27; *SD*^*norm*^=2.5, Franke et al. 2017) (*t*(570) = 15.40, *p <*.001), anxiety (*M* = 3.67; *SD* = 4.30, vs. *M*^*norm*^=1.09; *SD*^*norm*^=2.1, ibd.) (*t*(570) = 14.35, *p <*.001), and somatization (*M* = 2.23, *SD* = 3.00, vs. *M*^*norm*^=0.70; *SD*^*norm*^=14.8, ibd.) (*t*(570) = 12.18, *p <*.001). Moreover, SMA reported still higher scores on all three scales of the BSI-18: depression (SMA *M* = 7.93, *SD* = 5.25, NSMA *M* = 3.06, *SD* = 3.59) (*U* = 11,606, *Z* = -10.47, *p <*.001), anxiety (SMA *M* = 6.18, *SD* = 5.34, NSMA *M* = 2.92, *SD* = 3.61) (*U* = 16,841, *Z* = -7.31, *p <*.001), and somatization (SMA *M* = 3.60, *SD* = 4.02, NSMA *M* = 1.82, *SD* = 2.48) (*U* = 19,730, *Z* = -5.64, *p <*.001) than NSMA. Spitzer et al. (2011) reported BSI-18 patient scores relatively close to SMA scores for depression (mean scores ranging from 6.17 to 11.61) and anxiety (mean scores ranging from 6.26 to 9.51), but not for somatization (mean scores ranging from 6.47 to 6.90). It can therefore be assumed that students in this sample are generally more mentally stressed, with students who are addicted to social media being particularly mentally stressed. This finding could be explained due to the high distress and burden in the early phase of the COVID19 pandemic.

### Two-step cluster analysis

The two-step cluster analysis suggested a three-cluster solution as the most appropriate fit. Evaluation of the centroids of continuous variables (Table 4) and frequencies of the categorical cluster composition (Table 5) result in the following clusters:

**Table 4:** Centroids of CA/mean values for each continuous variable by cluster

Cluster	z-scores		z-scores		z-scores		z-scores		z-scores			
	Extraversion		Neuroticism		Depression		Anxiety		Somatization		Age	
	Mean	SD	Mean	SD	Mean	SD	Mean	SD	Mean	SD	Mean	SD
ACA	0.162	0.899	− 0.083	0.926	− 0.139	0.806	− 0.141	0.744	− 0.015	0.870	24.06	6.297
SA	0.172	1.028	− 0.263	1.131	− 0.421	0.584	− 0.362	0.630	− 0.288	0.576	23.66	4.149
AVA	− 0.243	1.011	0.250	0.925	0.347	1.198	0.320	1.203	0.164	1.205	23.35	4.477
Comb.	− 0.005	1.00	0.011	1.003	− 0.006	1.001	− 0.003	0.985	− 0.009	0.986	23.66	5.067

**Table 5:** Categorical cluster composition. Frequency (F) of each categorical variable’s levels by cluster

Cluster	Secure attachment		Rather secure attachment		Avoidant closed a.		Ambivalent closed a.		Ambivalent clingy a.		Addiction		Gender	
	0	1	0	1	0	1	0	1	0	1	0	1	W	M
	F		F		F		F		F		F		F	
ACA	178	0	178	0	178	0	178	0	0	178	141	37	125	53
SA	98	42	95	45	87	53	140	0	140	0	130	10	92	48
AVA	231	0	231	0	231	0	0	231	231	0	155	76	150	81
Comb.	507	42	504	45	496	53	318	231	371	178	426	123	367	182

The **Cluster ambivalent clingy attachment** (ACA) (*N* = 178) is relatively balanced in terms of extraversion, neuroticism, depression, anxiety, and somatization. They are uniquely characterized by the ambivalent clingy attachment style with a balanced representation of social media dependence.

The **Cluster secure attachment** (SA) (*N* = 140) is characterized by individuals who are slightly extroverted, less neurotic, and show lower levels of depression, anxiety, and somatization. This cluster stands out due to its representation of secure and rather secure attachment styles and has the lowest proportion of individuals who are addicted to social media.

The **Cluster ambivalent closed attachment** (AVA) (*N* = 231) is slightly introverted, more neurotic, and exhibits higher levels of depression and anxiety. Participants of this cluster are exclusively of the ambivalent closed attachment style, and a significant portion seems more susceptible to social media addiction.

For the validation of the derived clustering solution, the Bayesian Information Criterion (BIC) was employed as a model selection criterion to identify the optimal number of clusters. The BIC is advantageous in balancing the goodness of fit of the model against its complexity, penalizing models with more parameters to avoid overfitting. Various numbers of clusters were considered, ranging from 1 to 15, and the corresponding BIC values were calculated for each cluster solution. Table 6 presents the BIC values obtained for different cluster solutions. The BIC drops substantially from 1 cluster to 2 clusters, indicated by a change of -1180.384. There is a smaller but still notable drop from 2 clusters to 3 clusters, with a change of -605.464. After 3 clusters, the BIC drops more slowly, with smaller changes for each additional cluster. Even if the ratio for the change from 2 to 3 clusters is 0.512, the ratio of distance measures that indicates how distinct the clusters are from each other is for the 3-cluster solution still 1.780, which suggests that the 3-cluster solution is equally well-defined compared to the 2-cluster solution. Given this information, we opt for the 3-cluster solution, since the BIC drops more slowly beyond this point, suggesting diminishing returns in terms of model fit as more clusters are added and the 3-cluster solution offers a sufficient granular segmentation. The distribution of age (Table 4) and gender (Table 5) was relatively even.

**Table 6:** BIC values and changes for different cluster solutions

Number of Clusters	Schwarz Bayesian Criterion (BIC)	BIC-Change^a^	Ratio of BIC-Cange^b^	Ratio of Disctance Measures^c^
1	6050.957			
2	4870.574	-1180.384	1.000	1.793
3	4265.109	-605.465	0.513	1.781
4	3977.668	-287.441	0.244	1.335
5	3792.535	-185.133	0.157	1.230
6	3664.418	-128.117	0.109	1.023
7	3541.860	-122.559	0.104	1.050
8	3430.772	-111.088	0.094	1.220
9	3361.296	-69.476	0.059	1.565
10	3360.201	-1.095	0.001	1.298
11	3386.852	26.651	− 0.023	1.067
12	3419.344	32.492	− 0.028	1.044
13	3455.556	36.212	− 0.031	1.046
14	3495.457	39.901	− 0.034	1.131
15	3544.605	49.148	− 0.042	1.085

*Notes*. (a) Changes are derived from the previous number of clusters in the table. (b) The change ratios are relative to the change for the two cluster solutions. (c) The ratios for distance measures are based on the current number of clusters compared to the previous number of clusters

## Discussion

### Principal results

This study aimed to examine social media addiction with a focus on differences in attachment style, mental distress, and personality between students with and without social media addiction. For personality, it was shown that SMA had significantly higher values on the neuroticism dimension than NSMA, but they did not differ in the extraversion dimension. Thus, SMA can be classified as more emotionally unstable in comparison with NSMA. Further, SMA scored significantly higher on all three levels—depressiveness, anxiety, and somatization—than the group of NSMA, i.e., social media addicted users are comparatively more mentally stressed. At least for attachment style, the assumption that SMA are more likely to show an insecure attachment was confirmed here. In more detail, most SMA displayed an ambivalent closed attachment style. Two-step cluster analysis yielded a holistic insight into the collective grouping of cases by these variables. It corroborated the findings of the univariate analyses, revealing three predominant clusters, chiefly characterized by three attachment styles and varying levels of social media addiction: (a) secure attachment, predominantly associated with fewer instances of social media addiction and lower prevalence of mental health problems; (b) ambivalent closed attachment, typically marked by a higher frequency of social media addiction and elevated levels of mental health problems; and (c) ambivalent clingy attachment, presenting a moderate incidence of social media addiction and a relatively balanced mental health profile.

### Social media usage

Prevalence rate of social media addiction (22.8%) lies within the literature reported prevalence of the used instrument (BSMAS), since Chen et al. [10] specify < 10–40% for the BSMAS. SMA differ from NSMA in their usage of social media, exhibiting higher values in usage frequency, time spent, and perceived importance. Notably, SMA are more active on image-oriented services such as “Instagram” and “Pinterest”. They also report higher levels of “comparison with others”, “dissatisfaction with their own body (especially when exposed to idealized online images)”, and “negative self-esteem when interacting with influencers”. This suggests that SMA may experience heightened negative body awareness when comparing themselves to online images. Moreover, SMA use social media for a broader range of purposes compared to NSMA.

### Personality

SMA scored significantly higher on the neuroticism dimension than NSMA, suggesting that they tend to be more emotionally unstable and easily irritable. Conversely, no difference was observed in the extraversion dimension. Previous research supports the idea that internet-related addictions are linked to higher scores on the neuroticism dimension. Blackwell et al. [27] demonstrated that neuroticism predicts social media use. Moreover, a study by Müller [55] suggests that Internet addiction correlates with increased neuroticism scores. Interestingly, individuals with elevated neuroticism scores associate Internet topics with significantly stronger positive arousal compared to a healthy control group [56]. Social media addiction has also been positively linked to neuroticism [27, 28], and individuals scoring high on this trait are drawn to social networks as they offer recognition and validation [27]. Marengo et al. [28] align with our findings by not observing a relationship between social media addiction and extraversion. The contrasting findings presented by Kuss and Griffiths [29] relate extraversion more to older individuals and openness more to younger ones. Given our primary focus on younger participants, our results are consistent with these observations.

### Mental distress

SMA display significantly higher values for depression, anxiety, and somatization compared to NSMA, even considering the evident distress in the overall sample. This suggests that SMA may be mentally more strained than NSMA. Consequently, further evidence for the connection between mental disorders and internet-related addictions in terms of comorbidity was found in the present study. This augments the extant research on depression, anxiety, and internet addiction. Kırcaburun [57] also identified a significant positive relationship between depressive symptoms, internet use, and social media addiction. In his study, the level of depression in adolescents was indirectly influenced by social media addiction; addicts spent more time online, amplifying the risk of depressive symptoms. Similarly, Wu et al. [58] found that internet addiction correlates with depression in adolescents, exerting direct, mediated, and moderating effects on depression levels. For anxiety, there’s also documented evidence of a positive association with problematic social media consumption. Baltaci [23] highlighted social anxiety as a predictor for social media addiction among university students. Other studies have shown a positive correlation between internet addiction and general anxiety levels in students [59, 60]. As for somatization, there’s a documented positive correlation with internet addiction in adolescents [61–63]. Research on somatization and smartphone addiction is somewhat limited [63]. Results here confirm the positive correlation adding to this research corpus also heightened somatic symptomatology in social media addicted students.

### Attachment

Users with an insecure attachment style are significantly more likely to exhibit social media addiction than those with a secure attachment style. These findings align with a substantial body of research that establishes a connection between insecure attachment styles and internet-related addictions. A systematic review has provided evidence linking insecure attachment styles with both internet addiction in general and social media addiction in particular [64]. Moreover, certain studies suggest that difficulties in relational behavior or the presence of insecure attachment styles can act as risk factors for smartphone addiction. For instance, Baek et al. [65] identified a correlation between attachment behavior (specifically internalization problems) and smartphone usage. Other research [66, 67] has indicated a mediating effect of attachment style on smartphone addiction. Anxiously attached individuals showed patterns of self-regulation that directly influenced their susceptibility to smartphone addiction. While a secure attachment style offered a protective effect, an anxious attachment style increased vulnerability to addiction. In contrast, an avoidant attachment style didn’t significantly influence addiction development.

For social media addiction, several studies have highlighted its relationship with attachment. For instance, Hart et al. [37] demonstrated a link between dysfunctional attachment qualities and problematic social media use. A study involving Turkish students revealed that insecure attachment styles might serve as risk factors for social media addiction [38]. Conversely, secure attachment and high self-esteem can act as protective factors against such addiction [38]. Numerous studies have established a connection between an anxious attachment style and both heavy social media use [39–41] and addiction to it [42]. Specifically, Yaakobi and Goldenberg [43] identified a positive correlation between an anxious attachment style and the amount of time spent on social media. This same study found that an anxious attachment style negatively predicts the number of online friends. Oldmeadow et al. [41] also discovered a relationship between anxious attachment and seeking comfort on Facebook, noting an increase in Facebook usage, especially during negative emotional states.

Currently, no studies explore the relationship between an ambivalent closed attachment style and social media addiction. However, the findings in this study indicate that an ambivalent closed attachment style is significantly associated with social media addiction more frequently. These results are consistent with previous data suggesting this style is prevalent for internet-related addictions, as observed in the context of both smartphone [34] and internet [36] addictions. According to Höger and Buschkämper [53], individuals with an ambivalent attachment style exhibit an increased need for attention and concurrently face heightened acceptance issues. This pattern suggests heightened anxiety and a secondary hyperactivating (ambivalent) strategy (ibid.). It’s plausible that the social-compensatory component is particularly influential in this context when it comes to social media [34]. Individuals with an ambivalent-closed attachment style might turn to online platforms, especially social media, to mitigate their interpersonal relationship deficits (ibid.). The anonymity afforded by the internet allows a new representation of the self to be created, helping this group to compensate for feared problems of acceptance (ibid.). Based on the data, it appears this new representation of the self is often facilitated through image-focused platforms like “Instagram” and “Pinterest”. However, this may inadvertently expose SMA users to the pitfalls of social media, such as body dissatisfaction and reduced self-esteem when interacting with influencers. This dynamic could exacerbate their acceptance issues, perpetuating a detrimental cycle.

The ambivalent clinging and closed attachment styles differ primarily in their perceived willingness to open up. The former demonstrates a moderate willingness, allowing for the expression of strong attachment needs associated with the hyperactivated attachment system, while the latter exhibits a notably low willingness to open [53]. The findings presented in this study indicate that the degree of openness (for attachment) may play a crucial role in determining the severity of problematic user behavior. Specifically, the more receptive a user is to attachment, the less likely they are to exhibit addictive behaviors. Cluster analysis supports this interpretation. It identified three clusters with varying susceptibilities to social media addiction: those with secure attachment exhibit the lowest likelihood, those with ambivalent clingy attachment have a medium likelihood, and those with ambivalent closed attachment display the highest likelihood. This potential correlation warrants further exploration in subsequent research. Moreover, given that a mediating effect of mentalization between attachment style and both emotion dysregulation [68] and psychopathology [69] has been demonstrated, future research should delve deeper into exploring the relationships between mentalization, attachment style, and internet-related addictions.

### Limitations

It should be noted that the data are based on self-reporting in an online survey. Response rate is comparable with other online-survey studies [70]. So, possible self-selection processes could be of importance since online surveys are prone to an inherent selection bias. Social media users may find it appealing to participate for trying to relativize the negative image of social media addiction. Further, the sample is due to the narrow age distribution and educational level not representative. Even if cluster analysis shows no noteworthy age distribution for the clusters, future research should collect sufficient case number for each age group or limit age to a homogenous group. Female students contributed disproportionately here. Which in turn can affect the prevalence of social media addiction since there is evidence that women are more prone to social media addiction [8]. Though, this gender bias has been frequently observed in online surveys [71]. Cluster analysis did not reveal any conspicuous distribution for gender either. Altogether, future studies with a broader recruitment strategy may provide more representative data and confirm discussed results. Further, it could be discussed that the design of the study is cross-sectional. Since there is evidence for differences in age, at least for personality dimensions, comparison of two points in time or more can corroborate data or reduce it to differences in generation cohort. Furthermore, since mental health is a key variable, future studies should check psychiatric history of participants.

## Conclusions

This study enhances our understanding of how specific attachment problems could contribute to the development of social media addiction, reaffirming findings related to internet and smartphone addiction. It reveals that an avoidant closed attachment style, characterized by a pronounced need for attention, acceptance issues, and notably low openness for attachment, is frequently associated with this addiction. Such a deficit in openness may prompt compensatory behavior to satiate the intensive need for attention in the manageable environment of the digital world, where any conversation can be terminated with a click. This intense attention-seeking behavior seems to find satisfaction through image-centric services on social media, instigating negative comparative processes with others and potentially reinforcing acceptance issues in a self-perpetuating cycle, with mental stress being a substantial correlate.

To break this cycle, therapeutic interventions should consider these interrelations and specifically target critical areas. This could include conducting a thorough media anamnesis, educating about the effects of image-focused services and comparative processes, and establishing a robust and consistent therapeutic alliance—a cornerstone of successful addiction treatment [34]. The incorporation of attachment-oriented strategies is vital, as attachment-related aspects have yet to be integrated into existing internet addiction treatment protocols [34, 36]. In addition, since research showed a good impact of whole school attachment-based interventions [72], prevention programs to combat digital addictions in schools and universities should also include content that promotes secure attachment behavior, especially to young people with a high need for attention, acceptance issues, and notably low openness for attachment. Beyond individual treatment, the implementation of these strategies has the potential to foster a healthier approach to digital media usage across society, thereby contributing to a more informed and mindful engagement with social media platforms, which can finally lead to a reduction in the prevalence and impact of social media addiction on a broader scale.

## Data Availability

The raw data supporting the conclusions of this article will be made available by the authors, without undue reservation.

## Abbreviations

BFI-10: Big Five Inventory
BFPE: Bielefelder Fragebogen zu Partnerschaftserwartungen
BSI-18: Brief Symptom Inventory
BSMAS: Bergen Social Media Addiction Scale
NSMA: Not Social Media Addicted
SMA: Social Media Addicted
